# Epidermal Growth Factor is Effective in the Treatment of Diabetic Foot Ulcers: Meta-Analysis and Systematic Review

**DOI:** 10.3390/ijerph16142584

**Published:** 2019-07-19

**Authors:** Thien Quoc Bui, Quoc Van Phu Bui, Dávid Németh, Péter Hegyi, Zsolt Szakács, Zoltán Rumbus, Barbara Tóth, Gabriella Emri, Andrea Párniczky, Patricia Sarlós, Orsolya Varga

**Affiliations:** 1 Department of Preventive Medicine, Faculty of Medicine, University of Debrecen, 4028 Debrecen, Hungary; 2 Department of Internal Medicine, Faculty of Medicine, Pham Ngoc Thach University of Medicine, Ho Chi Minh city 70000, Vietnam; 3 Institute for Translational Medicine, Medical School, University of Pécs, 7624 Pécs, Hungary; 4 Department of Pharmacognosy, Faculty of Pharmacy, University of Szeged, 6720 Szeged, Hungary; 5 Department of Dermatology, Faculty of Medicine, University of Debrecen, 4032 Debrecen, Hungary; 6 Heim Pál National Institute of Pediatrics, 1089 Budapest, Hungary; 7 Division of Gastroenterology, First Department of Medicine, University of Pécs, Medical School, 7624 Pécs, Hungary; 8 Department of Preventive Medicine, Faculty of Public Health, University of Debrecen, 4028 Debrecen, Hungary

**Keywords:** recombinant human epidermal growth factor, placebo, diabetic mellitus, diabetic foot ulcer, meta-analysis

## Abstract

Diabetic foot ulcers (DFUs) are one the common complications of diabetes mellitus. Many trials were performed to evaluate the effect of recombinant human epidermal growth factor (rhEGF) in healing DFUs. This meta-analysis was performed to synthesize the evidence of rhEGF treatment in DFUs in comparison to placebo. Databases included for the search were PubMed, EMBASE, the Cochrane Library, Web of Science, EBSCOhost, ScienceDirect, and Scopus (up to January 2019). The outcome of interest was the complete healing rate of DFUs. We performed random effects meta-analysis stratified by the types of administration route (intralesional injection and topical apply) by calculating the odds ratios (OR) and 95% confidence interval (95% CI). A total of six studies involving 530 patients were eligible for analysis. The combined OR (intralesional injection and topical apply) was 4.005 (95% CI: (2.248; 7.135), *p* < 0.001). The ORs for intralesional injection and topical application were 3.599 (95% CI: (1.213; 10.677), *p* = 0.021) and 4.176 (95% CI: (2.112; 8.256), *p* < 0.001), respectively. Statistical heterogeneity might not be important in overall treatment (I^2^ = 15.17, *p* = 0.317) and both of the subgroups (I^2^: 24.56, *p* = 0.25 and I^2^: 33.26, *p* = 0.213, respectively). Our results support the use of rhEGF in the treatment of DFUs.

## 1. Introduction

Diabetes mellitus (DM) is caused by a deficiency of either insulin production or insulin function. Untreated or inadequately treated DM results in many complications, such as micro-vasculopathy (including retinopathy, nephropathy, and neuropathy) and macro-vasculopathy (including cardiovascular disease and insufficient blood flow to lower limbs) [1]. Neuropathy is the main etiology of diabetic foot ulcers (DFUs). The mechanism of this includes direct damage to the nerve by hyperglycemia and a decrease in blood flow to the nerves by damaging small blood vessels. This nerve damage often results in sensory loss and damage to lower limbs which the patient is unaware of [2]. Based on some estimations made in 2017, DM affects 451 million people aged between 18 and 82 worldwide, with a global expenditure on DM of 850 billion USD [3,4]. The global prevalence of diabetic foot ulcers was 6.3%, with North America having the highest (13%) and Oceania having the lowest values (3%). The prevalence was 5.5%, 5.1%, and 7.2% in Asia, Europe, and Africa, respectively [3]. Indeed, DM is one of the leading causes of non-traumatic lower extremity amputation.

According to the National Institute for Health Care Excellence guidelines (NICE guidelines (NG19) 2016) and Wound Healing Society (WHS) guidelines, the treatment for DFUs remains to be the standard of care, which includes offloading, infection control, ischemia control, wound debridement, and wound dressing [5,6]. However, in the NICE guidelines, it is emphasized that electrical stimulation therapy, autologous platelet-rich plasma, regenerative wound matrices, dalteparin, and growth factors (including granulocyte colony-stimulating factor (G-CSF), platelet-derived growth factor (PDGF), epidermal growth factor (EGF), and transforming growth factor beta (TGF-β)) are not recommended, unless as part of a clinical trial.

Based on the results of a systematic review assessing a variety of growth factor functions, growth factors may contribute to achieving the complete healing of foot ulcers in patients with DM [7]. EGF, previously known as urogastrone, stimulates cell proliferation, differentiation, and survival by binding to the EGF receptor [8,9]. By exploiting this function, recombinant EGF is manufactured and marketed under the brand name Heberprot-P® 25/75 (Heber Biotec, S.A. La Habana, Cuba) -intralesional injection form, Regen-D™ 150 (Bharat Biotech, Hyderabad, India) -ointment form and Easyef 0.005% (Daewoong Pharmaceutical Co., Seoul, South Korea) -spray form. Our hypothesis is that the application of rhEGF will facilitate the healing process of DFUs.

## 2. Methodology

The meta-analysis was reported according to the Preferred Reporting Items for Systematic Reviews and Meta-Analysis (PRISMA) statement, and was registered in the international prospective register of systematic reviews (PROSPERO) with registration number CRD42019126404.

### 2.1. Information Source and Search Strategy

The literature search was performed in PubMed, EMBASE, the Cochrane Central Register of Controlled Trials (CENTRAL), Web of Science, EBSCOhost, ScienceDirect, and Scopus databases until 10 January 2019. The key terms were diabetic foot, diabetic foot ulcer, diabetic ulcer, diabetes and recombinant human epidermal growth factor, rhEGF, epidermal growth factor, EGF, urogastron*. Only an English language filter was applied. All the relevant articles included for the analysis were imported and selected manually on Endnote x9 software. 

### 2.2. Eligibility, Study Selection, and Data Extraction

Randomized, placebo-controlled trials evaluating the effects of rhEGF administration (intralesional injection, topical-gel, cream) in patients with DFUs were included. 

Title, abstract, and full text base screening were performed by three independent investigators (T.Q.B, Q.V.P.B, and O.V). One investigator (T.Q.B) extracted the data from eligible studies and two investigators (Q.V.P.B, O.V) checked for data accuracy.

The following data were extracted from the eligible studies: name of the first author, year of publication, study design, demographic information, patient data, ulcer baseline data, interventional data, and outcome data, including primary outcomes—such as complete healing rate, ulcer size change, granulation response, and days to complete healing—and secondary outcomes—healing velocity, hospital stay, days to 50% wound size reduction, and percentage of patients with adverse events.

### 2.3. Data Synthesis and Analysis

Statistical analysis was performed using Comprehensive Meta-Analysis (version 3.3, Biostat, INC., Engelwood, MJ, USA). The primary outcome included in the analysis was the complete healing rate of DFUs. A subgroup analysis was performed, in which we divided the studies based on the administration route (intralesional injection versus topical). In addition, correlations between the complete healing rate and mode of drug administration were analyzed using logit regression, to decide the optimal route for applying rhEGF. The odds ratio (OR) and 95% confidence interval (95% CI) were calculated. When an OR was more than 1, it indicated that rhEGF treatment was favored over placebo. Heterogeneity between the studies in effect measures was assessed using both Q and I^2^ tests. Bases on Cochrane’s Handbook, a rough classification of the I^2^ index value is the following: low (0–40%), moderate (30–60%), substantial (50–90%), and considerable heterogeneity (75–100%) [10]. Depending on the similarity of the studies, a fixed or random effect model was used to handle heterogeneity. The effect sizes of the studies were visualized by a forest plot. 

### 2.4. Risk of Bias

Quality assessment was performed using the Cochrane Risk-of-Bias Assessment Tool for Randomized Control Trials. We assessed the random sequence generation (selection bias), allocation concealment (selection bias), blinding of participants and personnel (performance bias), blinding of outcome assessment (detection bias), incomplete outcome data (attrition bias), and other bias. For each domain, studies were judged to have either a high (red), unclear (yellow), or low (green) risk of bias. The risk of bias summary table and graph were regenerated by the RevMan software (version 5.3. Copenhagen: The Nordic Cochrane Center, The Cochrane Collaboration, 2014, Denmark). 

### 2.5. Publication Bias

Publication bias was assessed using funnel plot techniques. If asymmetry was found in a funnel plot, Begg’s rank test and Egger’s regression test would be used in advance.

### 2.6. Quality of Evidence

Quality of evidence on the complete healing rate was estimated using the Grading of Recommendations Assessment, Development, and Evaluation (GRADE) approach.

### 2.7. Sensitivity Analysis

Sensitivity analysis was performed by omitting any study (one by one) from the pooled analysis, then ORs on the complete healing rate were recalculated to study the impact of each individual study on the summary estimate.

## 3. Results

### 3.1. Study Selection

**Figure 1:**
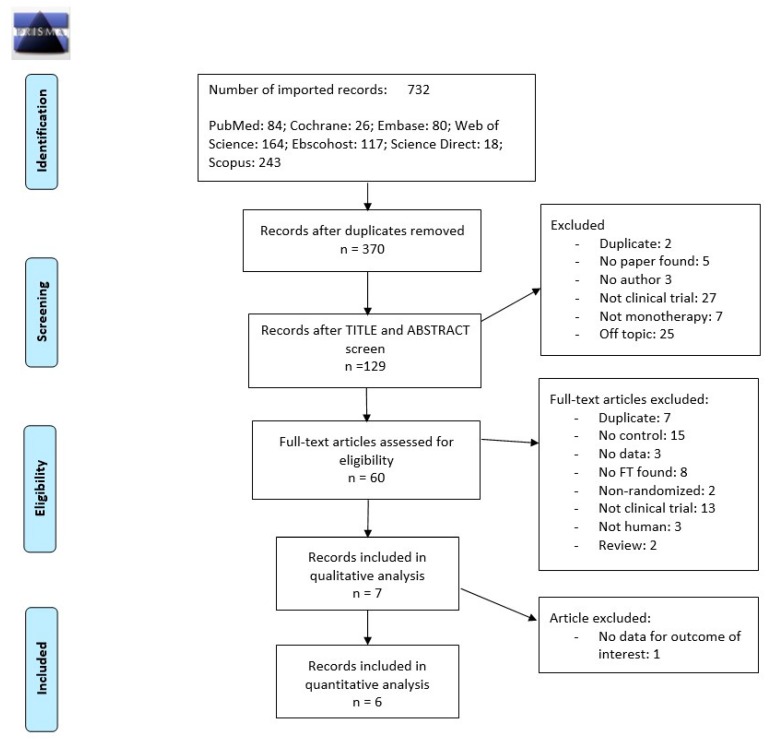
Flowchart illustrate the screening process for the inclusion of eligible studies for meta-analysis.

Eligible randomized controlled trials (RCTs) were selected according to the PRISMA flowchart presented in Figure 1. Seven studies were found to be eligible for qualitative analysis [11,12,13,14,15,16,17] and six of those were included in the quantitative synthesis (meta-analysis). The study of Xu et al. published in 2018 was excluded from our meta-analysis due to unavailable data on the complete healing rate; instead, the study mainly focused on healing time (wound healing initiation time, 50% wound surface healing time, and complete wound healing time) [17].

### 3.2. Study Characteristic

The basic characteristics of six studies, which included RCTs, for meta-analysis are shown in Table 1 and Table 2. The included studies were performed in five countries, Iran, India, China, Korea, Cuba, and Mexico, and were published between 2008 and 2018. In these studies, patients received either rhEGF or placebo intervention, in addition to standard diabetic foot management. The rhEGF and placebo treatments were administrated by intralesional injection or topical application. A total of 610 patients were recruited in all the studies and 540 patients completed the study periods, 307 of whom received rhEGF treatment and 233 of whom received placebo treatment. All patients were diagnosed with either type 1 or 2 DM and developed DFUs. Patients’ ages ranged from 18 to 82 years. The mean duration of DM ranged from 9.05 to 17.3 years and mean ulcer duration ranged from 4.3 to 59.7 weeks. Complete healing rate was studied throughout all the included studies. In addition, healing velocity, time to archive 50% reduction, time to complete ulcer healing, and granulation response were also studied in the study, as performed by Park et al. [16] and Xu et al. [17].

### 3.3. Odds Ratio of Complete Healing Rate with rhEGF Versus Placebo

**Figure 2:**
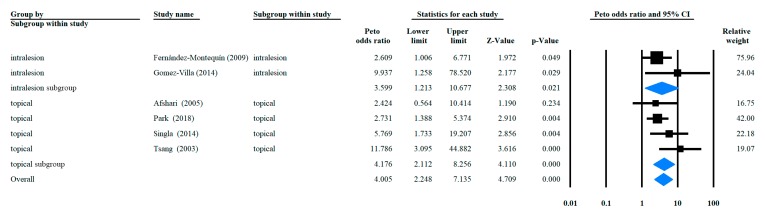
Forrest plot of complete healing rate categorized by different administration routes.

**Figure 3:**
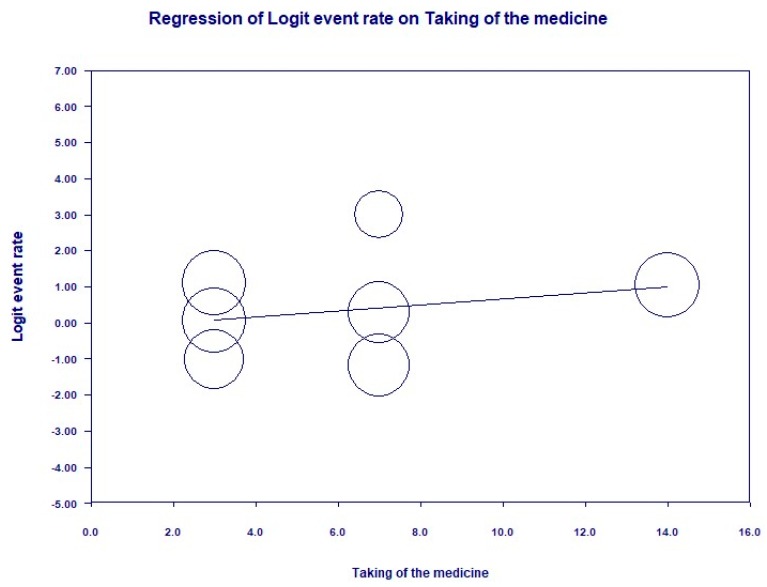
Logit regression on the correlation between the recombinant human epidermal growth factor (rhEGF) application frequency (horizontal axis—measured in weeks) and complete healing rate.

Statistical analysis results are shown in Figure 2. The overall OR was 4.005 (95% CI: (+2.248; +7.135), *p* < 0.001) and the subgroup ORs of intralesional injection and topical application were 3.599 (95% CI: (+1.213; +10.677), *p* = 0.021) and 4.176 (95% CI: (+2.112; +8.256), *p* < 0.001), respectively. Heterogeneity was found to exist overall (I^2^ = 15.17, *p* = 0.317) and in both the intralesional injection and topical application subgroups (I^2^ = 24.56, *p* = 0.25 and I^2^ = 33.26, *p* = 0.213, respectively). Meta regression analysis on the correlation between the rhEGF application frequency and complete healing rate resulted in a linear correlation, although it was insignificant (Figure 3).

### 3.4. Quality of Evidence

**Figure A1:**
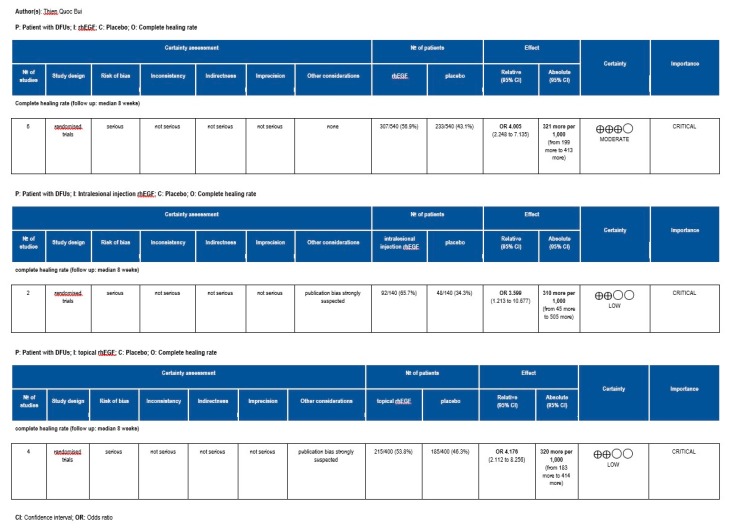
Grading of Recommendations Assessment, Development and Evaluation (GRADE) evidence profile.

In our meta-analysis, only randomized control trials were included. The overall quality of evidence on rhEGF treatment was moderate. Extra assessment was performed in correlation with different administration routes. The results of our GRADE analysis reported low evidence on using rhEGF via these routes. Downgrading factors commonly seen in our GRADE approach were publication bias and risk of bias. Details are shown in Appendix A, Figure A1. 

### 3.5. Risk of Bias Assessment

**Figure 4:**
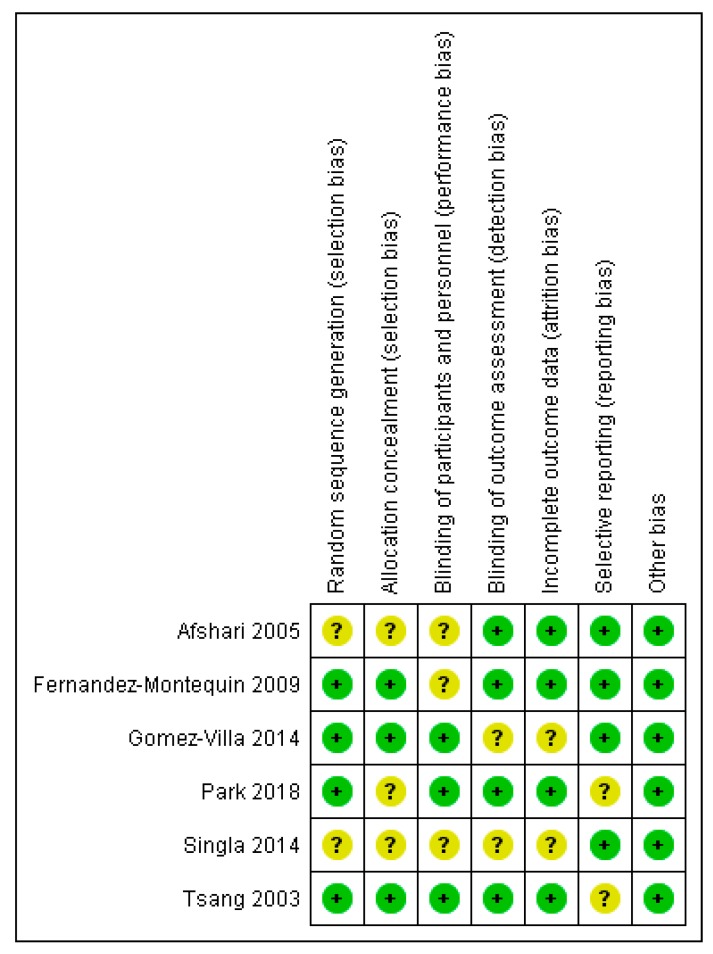
Risk of bias summary assessed for each eligible study.

**Figure 5:**
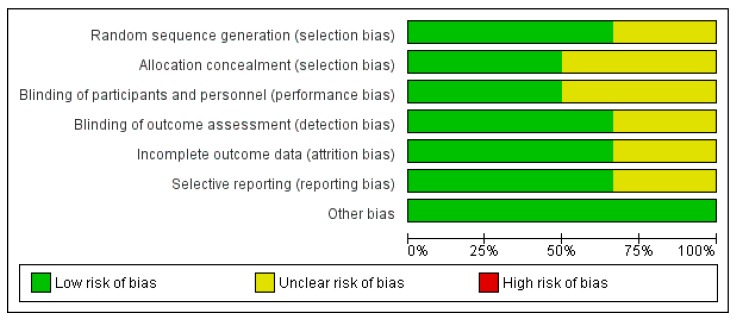
Risk of bias graph assessed for each criterion.

Details on the risk of bias assessment are illustrated in Figure 4 and Figure 5. The quality assessment was performed on a total of six studies, included in qualitative analysis, with the results showing mostly a low and unclear risk of bias. By converting the Cochrane Risk of Bias Tool to Agency for Healthcare Research and Quality (AHQR) standard, our risk of bias assessment reported a fair quality.

### 3.6. Publication Bias

**Figure 6:**
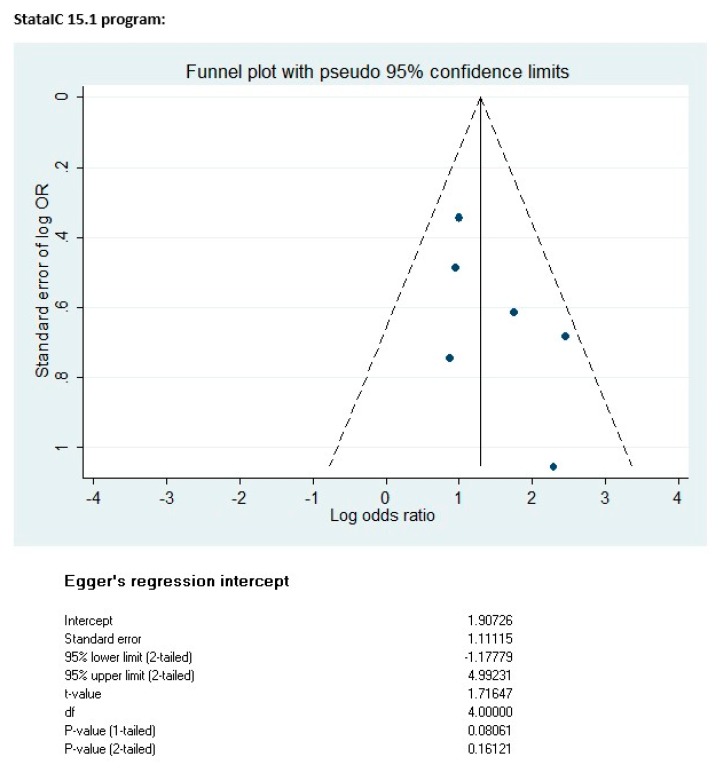
Funnel plot and Egger’s regression test on determining publication bias.

According to the Cochrane Handbook for Systematic Review of Interventions (version 5.1.0, Chichester: John Wiley & Sons, 2008, UK) a minimum number of 10 studies is recommended for assessing publication bias. Additionally, it has been shown previously that using only five or fewer studies is not sufficient for detecting publication bias asymmetry by using a funnel plot [18]. In this meta-analysis, the Stata/IC software (version 15.1, StataCorp LL, College Station, Texas, USA) was used to make a funnel plot and Egger’s regression test. Details are shown in Figure 6. The resulting *p*-value (two-tailed) of *p* = 0.161 means that publication bias was unlikely to occur. 

### 3.7. Sensitivity Analysis

**Figure 7:**
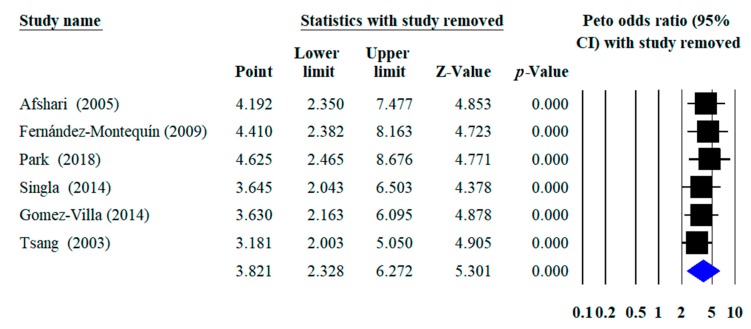
Sensitivity analysis with omitted studies and recalculated odds ratio.

Detail on sensitivity analysis are shown in Figure 7. The direction and magnitude of combined ORs with the respective omitted studies did not change significantly. 

## 4. Discussion 

This meta-analysis was designed to synthesize the currently available evidence on the usage of rhEGF in treating diabetic foot ulcers. According to the most recent guidelines of the Wound Healing Society (WHS), discussing the use of adjuvant on diabetic wounds, epidermal growth factor has not been proven to increase the proportion of wounds that heal or the healing rate of DFUs [6]. Seven studies were found to study the healing process of DFUs under rhEGF treatment in comparison to placebo. However, only six studies had data on the complete healing rate as their primary outcome, and these studies were pooled into our meta-analysis. Our results indicate that the use rhEGF together with standard wound care facilitates significantly improves healing rate compared to the placebo control in DFU treatments. This result is consistent with the findings of the previous meta-analysis [19]. However, in addition to the questions of the previous meta-analysis, a question with practical relevance has addressed the efficacy of rhEGF applied by different methods—intralesional injection or topical application. A subgroup analysis, categorized by the route of administration, was carried out to ensure the efficacy of rhEGF, which was maintained regardless of the administration route. Furthermore, we managed to determine the optimal way (intralesional injection or topical application) of delivering rhEGF in patients with DFUs. According to Berlanga-Acosta et al, parenteral administration of EGF on epithelial tissue provides the most effective healing for diabetic foot ulcers [20]. Our work could not confirm the aforementioned experiment result, however, this contradiction might be partly explained by the low number of eligible studies in each subgroup. Low heterogeneity (0–40%) was found in the overall and subgroup analyses. 

Although rhEGF was reported to significantly facilitate the healing process in DFUs, based on our meta-analysis result, the therapeutic management of DFUs is a multidisciplinary approach. Proper wound care is a key component during the treatment, but it is not sufficient. Many other factors, like offloading, infection control, ischemia control, and glycemic control, must also be taken into consideration, along with local wound care. Such complexity may limit the reproducibility of our results in every day clinical care.

An important strength of our meta-analysis is that with six clinical trials, we confirmed the efficacy of rhEGF in DFU treatment. Compared to the previous meta-analysis, our meta-analysis included two recent clinical trials, providing greater evidence. This is important, since the number of studies related to rhEGF and DFU treatment is still limited. The efficacy of rhEGF has been proven in both the intralesional injection and topical application routes. An insignificant linear correlation between application frequency and complete healing rate has been found, suggesting that increasing the frequency of applying rhEGF may result in a faster DFU healing rate. 

A possible limitation of our meta-analysis is that even though publication bias analysis was performed, with results indicating it unlikely to occur, the tests were underpowered by the low number of studies. The risk of bias assessment resulted in a fair quality, which may raise questions about the validity of the findings, as well as the design and execution of each individual study concerning rhEGF treatment. As a consequence, our work may either over- or underestimate the true effect of rhEGF in the treatment of DFUs during daily clinical practice. The GRADE approach on rhEGF treatment found low evidence. Thus, the effect estimate of rhEGF may be limited, and the true effect may be substantially different from the estimate effect. In our meta-analysis, important clinical aspects on medication, like optimal administration route, dose, and treatment duration, could not be covered, and thus they remain unclear. The amount of data on adverse effects was also limited, and hence we could not elicit the most common side effect experienced as a result of rhEGF treatment. In the recent advances in genetics, genes were also identified to play an essential role in DFU initiation and progression [21]. Therefore, there could be new genetic drug targets identified and new medicine developed for the novel treatment of DFUs.

## 5. Conclusions

Our meta-analysis supports the use of rhEGF in facilitating the healing process of DFUs. However, this conclusion should be considered with caution. The number of studies was limited, as well as numerous factors which were not taken into consideration when studying the efficacy of rhEGF. This reflects the need for more well-designed RCTs concerning rhEGF and DFU treatment. It is recommended that future designs include relevant data such as the patient glycemic profile. This is to ensure adequate glycemic control during rhEGF therapy. The patients’ general condition and co-morbidity are important clinical aspects. These are also recommended to be studied alongside rhEGF treatment, as they may interfere with the healing process of DFUs.

## Figures and Tables

**Table 1:** Study and patient characteristics with the corresponding type of intervention, from studies included for meta-analysis (NA: not available).

Author	Year of Publication	Study Design	Demography		Intervention Type	Route	Apply Frequency	Treatment Duration (weeks)	Patient Data			
			Country	Number of Centers					Number of Patients	Age ± SD (Years)	Type of DM	Duration of Diabetes ± SD (Years)
Tsang	2003	RCT	China	1	Actovecgin %	Topical	Once a day	12	19	64.37 ± 11.67	1 or 2	10.11 ± 8.29
					0.02 wt% rhEGF				21	68.76 ± 10.34		9.85 ± 7.79
					0.04 wt% rhEGF				21	62.24 ± 13.68		9.005 ± 6.19
Afshari	2005	RCT	Iran	1	1000 mg 1% sulfadiazine	Topical	Once a day	8	20	55.84	1 or 2	NA
					1 mg rhEGF in 1000 mg 1% sulfadiazine				30	58.8		NA
Fernández-Montequín	2009	RCT	Cuba	20	Placebo	Intralesional injection	Three times a week on alternate days	8	32	NA	1 or 2	15
					25 µ/g				33	NA		15
					75 µ/g				44	NA		19.5
Gomez-Villa	2014	RCT	Mexico	2	Placebo	Intralesional injection	Three times a week on alternate days	8	16	55.1 ± 10.6	1 or 2	15.3 ± 8.4
					75 µ/g				15	62.1 ± 12.8		17.3 ± 10
Singla	2014	RCT	India	1	Betadine	Topical	Once every two weeks	8	25	55.84	1 or 2	NA
					Urogastrone (rhEGF) gel 15 g				24	58.8		NA
Park	2018	RCT	Korean	6	Saline	Topical	Twice a day	12	72	59.31 ± 12.64	1 or 2	NA
					0.005% rhEGF spray				69	56.52 ± 12.71		NA
Xu	2018	RCT	China	1	Saline	Topical	Once a day	8	49	63 ± 4.56	1 or 2	NA
					40 IU/cm^2^				50	65 ± 3.65		NA

Abbreviations: rhEGF (recombinant human epidermal growth factor); SD (standard deviation); DM (diabetes mellitus); NA (not available).

**Table 2:** Patient ulcer data with the corresponding intervention type (NA: not available).

Author	Intervention Type	Route	Apply Frequency	Treatment Duration (weeks)	Ulcer Data						
					Location	Severity	Ischemia Level	Infection	Ulcer Area ± SD (cm^2^)	Ulcer Duration ± SD (Weeks)	Complete Healing Rate (%)
Tsang 2003	Actovecgin %	Topical	Once a day	12	Foot	Wagner 1 or 2	ABI ≥ 0.7	NA	3.48 ± 0.82	12 ± 15.47	42.1
	0.02 wt% rhEGF				Foot				3.4 ± 1.1	11.48 ± 14.68	57.14
	0.04 wt% rhEGF				Foot				2.78 ± 0.82	8.24 ± 5.55	95.3
Afshari 2005	1000 mg 1%sulfadiazine	Topical	Once a day	8	Foot	Wagner 1 or 2	ABI < 1: 49% participants	NA	103.4 ± 147.8	59.7 ± 55.5	10
	1 mg rhEGF in 1000 mg 1% sulfadiazine				Foot		ABI < 1: 50% participants		87.5 ± 103.2	42.9 ± 38.4	23.3
Fernández-Montequín 2009	placebo	Intralesional injection	Three times a week on alternate days	8	Foot	Wagner 3 or 4	Hemoglobin ≥100 g/L	NA	21.8	4.9	52.1
	25 µ/g				Foot				4.3	20.1	52.1
	75 µ/g				Foot				4.3	28.5	75.5
Gomez-Villa 2014	Placebo	Intralesional injection	Three times a week on alternate days	8	Foot	Texas 1, 2, and 3	ABI > 0.6	NA	11.9 ± 11.8	15.3 ± 8.4	0
	75 µ/g				Foot				19.2 ± 15.7	25.8 ± 44	23.5
Singla 2014	Betadine	Topical	Once every two weeks	8	Foot	Wagner 1 or 2	ABI ≥ 0.75	NA	NA	NA	12
	Urogastrone (rhEGF) gel 15 g				Foot				NA	NA	48
Park 2018	Saline	Topical	Twice a day	12	Foot	Wagner 1 or 2	TcPO2 ≥ 30 mmHg or palpable dorsalis pedis artery or posterior tibial artery	NA	2.35 ± 2.69	29.6 ± 60.2	50.6
	0.005% rhEGF spray				Foot				2.8 ± 3.72	38.48 ± 70.24	73.2
Xu 2018	Saline	Topical	Once a day	8	Foot	Wagner 1 or 2	NA	NA	2.35 ± 2.69	29.6 ± 60.2	NA
	40 IU/cm^2^				Foot				2.8 ± 3.72	70.24 ± 38.48	NA

Abbreviations: rhEGF (recombinant human epidermal growth factor); ABI (ankle-brachial index); TcPO2 (transcutaneous oxygen pressure); SD (standard deviation); NA (not available).

## References

[1] Papatheodorou K., Banach M., Bekiari E., Rizzo M., Edmonds M. (2018). Complications of Diabetes 2017. J. Diabetes Res..

[2] W.H.O. Diabetes Programme. https://www.who.int/diabetes/action_online/basics/en/index3.html.

[3] Zhang P., Lu J., Jing Y., Tang S., Zhu D., Bi Y. (2017). Global epidemiology of diabetic foot ulceration: A systematic review and meta-analysis (dagger). Ann. Med..

[4] Cho N.H., Shaw J.E., Karuranga S., Huang Y., da Rocha Fernandes J.D., Ohlrogge A.W., Malanda B. (2018). IDF Diabetes Atlas: Global estimates of diabetes prevalence for 2017 and projections for 2045. Diabetes Res. Clin. Pract..

[5] The National Institute for Health and Care Excellence (2016). Diabetic Foot Problems: Prevention and Management.

[6] Lavery L.A., Davis K.E., Berriman S.J., Braun L., Nichols A., Kim P.J., Margolis D., Peters E.J., Attinger C. (2016). WHS guidelines update: Diabetic foot ulcer treatment guidelines. Wound Repair Regen..

[7] Martí-Carvajal A.J., Gluud C., Nicola S., Simancas-Racines D., Reveiz L., Oliva P., Cedeño-Taborda J. (2015). Growth factors for treating diabetic foot ulcers. Cochrane Database Syst. Rev..

[8] Oka Y., Orth D.N. (1983). Human plasma epidermal growth factor/beta-urogastrone is associated with blood platelets. J. Clin. Investig..

[9] Goodlad R.A., Raja K.B., Peters T.J., Wright N.A. (1991). Effects of urogastrone-epidermal growth factor on intestinal brush border enzymes and mitotic activity. Gut.

[10] Higgins J.P.T., Altman D.G., Gøtzsche P.C., Jüni P., Moher D., Oxman A.D., Savovic J., Schulz K.F., Weeks L., Sterne J.A.C. (2011). The Cochrane Collaboration’s tool for assessing risk of bias in randomised trials. BMJ.

[11] Tsang M.W., Wong W.K., Hung C.S., Lai K.M., Tang W., Cheung E.Y., Kam G., Leung L., Chan C.W., Chu C.M. (2003). Human epidermal growth factor enhances healing of diabetic foot ulcers. Diabetes Care.

[12] Afshari M., Larijani B., Fadayee M., Darvishzadeh F., Ghahary A., Pajouhi M., Bastanhagh M.H., Baradar-Jalili R., Vassigh A.R. (2005). Efficacy of topical epidermal growth factor in healing diabetic foot ulcers. Therapy.

[13] Fernandez-Montequin J.I., Valenzuela-Silva C.M., Diaz O.G., Savigne W., Sancho-Soutelo N., Rivero-Fernandez F., Sanchez-Penton P., Morejon-Vega L., Artaza-Sanz H., Garcia-Herrera A. (2009). Intra-lesional injections of recombinant human epidermal growth factor promote granulation and healing in advanced diabetic foot ulcers: Multicenter, randomised, placebo-controlled, double-blind study. Int. Wound J..

[14] Gomez-Villa R., Aguilar-Rebolledo F., Lozano-Platonoff A., Teran-Soto J.M., Fabian-Victoriano M.R., Kresch-Tronik N.S., Garrido-Espindola X., Garcia-Solis A., Bondani-Guasti A., Bierzwinsky-Sneider G. (2014). Efficacy of intralesional recombinant human epidermal growth factor in diabetic foot ulcers in Mexican patients: A randomized double-blinded controlled trial. Wound Repair Regen..

[15] Singla S., Garg R., Kumar A., Gill C. (2014). Efficacy of topical application of beta urogastrone (recombinant human epidermal growth factor) in Wagner’s Grade 1 and 2 diabetic foot ulcers: Comparative analysis of 50 patients. J. Nat. Sci. Biol. Med..

[16] Park K.H., Han S.H., Hong J.P., Han S.K., Lee D.H., Kim B.S., Ahn J.H., Lee J.W. (2018). Topical epidermal growth factor spray for the treatment of chronic diabetic foot ulcers: A phase III multicenter, double-blind, randomized, placebo-controlled trial. Diabetes Res. Clin. Pract..

[17] Xu J., Min D., Guo G., Liao X., Fu Z. (2018). Experimental study of epidermal growth factor and acidic fibroblast growth factor in the treatment of diabetic foot wounds. Exp. Ther. Med..

[18] Sutton A.J., Duval S.J., Tweedie R.L., Abrams K.R., Jones D.R. (2000). Empirical assessment of effect of publication bias on meta-analyses. BMJ.

[19] Yang S., Geng Z., Ma K., Sun X., Fu X. (2016). Efficacy of Topical Recombinant Human Epidermal Growth Factor for Treatment of Diabetic Foot Ulcer: A Systematic Review and Meta-Analysis. Int. J. Low. Extrem. Wounds.

[20] Berlanga-Acosta J., Fernandez-Montequin J., Valdes-Perez C., Savigne-Gutierrez W., Mendoza-Mari Y., Garcia-Ojalvo A., Falcon-Cama V., Garcia Del Barco-Herrera D., Fernandez-Mayola M., Perez-Saad H. (2017). Diabetic Foot Ulcers and Epidermal Growth Factor: Revisiting the Local Delivery Route for a Successful Outcome. BioMed Res. Int..

[21] Laimer M. (2017). MAPK14 as candidate for genetic susceptibility to diabetic foot ulcer. Br. J. Dermatol..

